# Influence of age as a continuous variable on survival outcomes and treatment options in patients with upper thoracic esophageal carcinoma

**DOI:** 10.7150/jca.83490

**Published:** 2023-04-09

**Authors:** Yu Lin, Yuling Ye, Qiuyuan Huang, Binglin Zheng, Yong Yang, Yuanmei Chen, Weiguang Li, Hongqian Ke, Chuyan Lin, Yiping Zhang, Liyan Wang, Junqiang Chen, Yuanji Xu

**Affiliations:** 1 Department of Radiation Oncology, Clinical Oncology School of Fujian Medical University, Fujian Cancer Hospital, Fuzhou 350014, China; 2 Department of Radiation Oncology, Fujian Medical University Union Hospital, Fuzhou 350001, China; 3 Department of Thoracic Surgery, Clinical Oncology School of Fujian Medical University, Fujian Cancer Hosptial, Fuzhou 350014, China

**Keywords:** upper ESCC, age, prognosis, treatment

## Abstract

**Background:** This retrospective review of patients with upper thoracic esophageal squamous cell carcinoma (ESCC) analyzed the prognostic value of age, as a continuous variable, and offered insight into treatment options.

**Methods:** 568 upper ESCC patients underwent radical therapy between 2004 and 2016. Age as a continuous variable was entered into the Cox regression model with penalized spline (P-spline) analysis to investigate a correlation between age and survival outcomes.

**Results:** Before adjustment, P-spline regression revealed U-shaped survival curves. Sixty years was the optimal cut-off age for differences in overall and progression-free survival (OS, PFS). The cohort was divided into age groups ≤ 50, 51-69, and ≥ 70 years. Multivariate analyses showed no significant differences in either PFS or OS for patients aged ≤ 50 and 51-69 years. After adjusting for covariates, P-spline regression showed that the risk of mortality and disease progression increased with age, and ≥ 70 years was an unfavorable independent prognostic factor. For age ≥ 70 years, the OS and PFS associated with non-surgery was comparable to that of surgery. For patients younger, the OS and PFS of patients given surgery was significantly better than that of patients given non-surgery.

**Conclusion:** Age was an independent prognostic factor for upper ESCC. Patients ≥ 70 years achieved no significant survival benefit from surgery, but for those younger than 70 years surgery was the preferred treatment option.

## Introduction

Esophageal cancer (EC) is a common digestive tract malignancy, with the seventh leading incidence and sixth highest mortality rate worldwide in all malignancies 1. Esophageal squamous cell carcinoma (ESCC) is the dominant subtype of EC, accounting for approximately 90% of EC cases 2. Age is an acknowledged risk factor of ESCC, and 55 years is the watershed age above which incidence increases sharply 3. Among patients with ESCC, about 60% are 65 years old or older at the time of initial diagnosis 4. Even with advances in diagnostic imaging and therapeutic techniques, the age-standardized 5-year overall survival of EC in China from 2012 to 2015 was only 30.3% 5. The population of China is increasingly aging, and therefore the numbers of elderly patients with diagnosed esophageal malignancy can also be expected to increase. It is crucially important to evaluate the influence of age on survival outcomes and thus treatment decisions in ESCC. These questions are largely unanswered.

Age at diagnosis has consistently been a proven risk factor of prognosis in ESCC, and influences cancer treatment options 3,6. In 2010, Tanja et al. 7 reported that age was an independent adverse prognostic factor in patients with EC who were 70 years and older. In 2011, in a study by Donohoe et al. 8, patients with EC and not yet aged 35 years had a dismal prognosis. In 2015, Miyata et al. 9 showed that being 75 years or older was an independent prognostic factor in ESCC. Yet, according to more recent research, age was not a significant prognostic factor in EC. In 2017, Gao et al. 10 reported that being older than 75 years had little effect on patients' prognosis. Motoori et al. 11 in 2019 found that age was not a significant prognostic factor in ESCC, at any cutoff age. It is noteworthy that in the above studies the definition of elderly varied, and patients' ages were often dichotomized to simplify statistical analyses, explanations, and descriptions of results. However, a wealth of prognostic information can be missed by dichotomizing age, can lead to false-positive outcomes, and confound therapeutic strategies. Treating age as a continuous variable has become important when analyzing its influence on survival outcomes and treatment options in EC.

Upper thoracic ESCC is relatively uncommon, accounting for only 5% to 10% of all EC cases 12,13. The management of upper ESCC differs from that of tumors located in the mid and lower part of the esophagus. According to the guidelines of the National Comprehensive Cancer Network (NCCN), there is no consensus about the optimal treatment of upper EC 14. In addition, the prognostic factors for upper thoracic ESCC are largely unknown. Our recent study 15 showed that primary gross tumor volume (GTVp) was an independent prognostic factor for overall survival (OS) and progression-free survival (PFS), and could guide treatment in upper ESCC. In that study 15, age was dichotomized using 60 years as the cutoff, and as such it was not a significant factor for predicting survival. This 60-year cutoff had been postulated based on our common clinical experience. We have since considered that treating age as a continuous variable may be more reliable when analyzing its influence on prognosis. A recent study to predict survival differences in patients with extranodal nasal-type NK/T-cell lymphoma determined the optimal age cutoff via multivariate Cox regression analysis using P-splines in smoothHR 16. As this analytic method is designed to investigate an association between a continuous variable and survival rates, it may be a more accurate way to explore the genuine prognostic value of age in ESCC. The determination should provide insights into treatment options.

The present study investigated the prognostic value of age in upper ESCC using P-spline regression analyses. It further explored the effect of age on treatment selection. This should establish the prognostic importance of age and provide a clinical reference for individualized treatment of patients with upper ESCC.

## Methods

### Study population

The Clinical Oncology School of Fujian Medical University, Fujian Cancer Hospital Institutional Board Review approved this retrospective study (No. SQ2020-063-01). Each patient provided written informed consent before treatment. Patient records and information were anonymized prior to analysis. The data was collected from the records of patients with diagnosed ESCC and treated in our hospital from February 2004 to December 2016.

For inclusion in the present analysis, patients fulfilled the following criteria: given no prior treatment; with pathologically proven ESCC without distant metastases; tumor located at the upper thoracic region (as defined by the NCCN guideline 14); Eastern Cooperative Oncology Group performance status (ECOG/PS) ≤ 3; completed pretreatment in accordance with the institutional protocol 17; and TNM (tumor-node-metastasis) staging conformed to the guidelines for EC of the eighth edition of the Union for International Cancer Control/American Joint Committee on Cancer (UICC/AJCC). The GTVp was contoured by two experienced thoracic radiotherapists expert in interpreting chest computed tomography images before treatment, as described in our previous study 18. Potential subjects were excluded if they had recurrent disease; secondary malignancies; or another tumor located in the cervical, middle, or lower third thoracic esophagus.

A total of 568 patients with upper ESCC were included in this analysis. Patients were classified based on age into 3 groups: ≤ 50, 51-69, and ≥ 70 years, with 81, 408, and 79 patients, respectively. Subjects were further stratified depending on the modality of treatment, into a surgery or non-surgery group. The surgery group (n = 352) comprised patients given radical surgery; or pre- or postoperative radiotherapy as well as radical surgery. The non-surgery group (n = 216) received definitive radiotherapy, with or without concurrent chemotherapy. Patients in each group may or may not have also received pre- or postoperative chemotherapy, either neoadjuvant or adjuvant chemotherapy of radiotherapy.

### Treatments

Surgical treatment included radical resection of local tumors and regional lymph nodes, which was conducted by institutional procedures described previously 19. Radiotherapy technology included 3-dimensional conformal radiation therapy or intensity-modulated radiation therapy. The definitive 20, pre-[17] and post-operative 21 radiotherapy treatments, including the targets, target dose, and dose limitations to organs at risk have been described as cited. The median radiation dose to the targets for definitive, pre-, and post-operative radiotherapy were, respectively, 61.5 (range, 50.0-67.2), 40.0 (36.0-50.0), and 50.0 (40.0-63.0) Gy with conventional fractions.

Chemotherapy treatments were platinum-based, and administered to 370 (65.1%) of the patients, of whom 207 (55.9%) and 163 (44.1%) were in the surgery and non-surgery group, respectively.

### Surveillance

The surveillance schedule for patients was performed as in our previous study 17. In short, patients were followed-up every 3 months for the first 2 years; every 6 months for years 3 to 5; and annually thereafter. The final follow-up date was 19 December 2019. The median surveillance time was 41.5 months. The primary endpoint was overall survival (OS), which was calculated from the start of diagnosis to the date of death or last follow-up. The secondary endpoint was progression-free survival (PFS), defined as the date of diagnosis to the date of death, local and/or regional relapse, distant metastasis, or last follow-up. The follow-up examinations included routine laboratory test; neck/chest/abdomen computed tomography (CT) scan; cervical/abdominal lymph node ultrasound; barium swallow; and/or positron emission tomography (PET)/CT. If suspicious recurrent lesions were found through imaging, biopsy was attempted.

### Statistical analyses

A multivariate Cox proportional hazards regression model with penalized spline (P-spline) regression was applied to evaluate the optimal cutoff age indicating a survival difference. This method enabled a nonlinear association between age and OS or PFS. P-spline was performed using the smoothHR package in R, version 4.0.3 22. P-spline presents an adaptable model to determine the correlation of age with the natural logarithm of a hazard ratio (HR) without prior knowledge of the classification of association, while adjusting for the influences of covariates. A histogram was created using HiPlot, version v0.1.1. Other data were analyzed using SPSS, version 26.0 (IBM, Armonk, NY, USA). The clinical features of the different subgroups were compared with Crosstabs. OS and PFS rates were calculated using the Kaplan-Meier method, and subgroups were compared with the log-rank test. Multivariate analyses of clinical features were employed to determine independent prognostic factors for OS and PFS via Cox proportional hazards regression.

## Results

### Age distribution and clinical characteristics

The final study population comprised 568 patients (391 men, with a gender ratio 2.2 to 1; Table 1). The median age at diagnosis was 60 years (range, 38-90 y).

For the entire cohort, the 5-year OS and PFS rates were 44.6% and 40.2%, respectively. The 5-year OS rates of the surgery and non-surgery groups were 55.7% and 50.6%; the 5-year PFS rates were 37.0 and 29.6%. Ultimately, 319 patients died of the following: 105 of distant metastasis; 134 of local or regional relapse; and 11 and 69 of other medical or unknown causes. There were 148 patients who experienced tumor relapse (43, 86, and 19 of local, regional, or combined relapse). Overall, 113 (19.9%) patients developed distant metastasis.

### Optimal cut-off for differences in survival before adjustment

**Figure 1:**
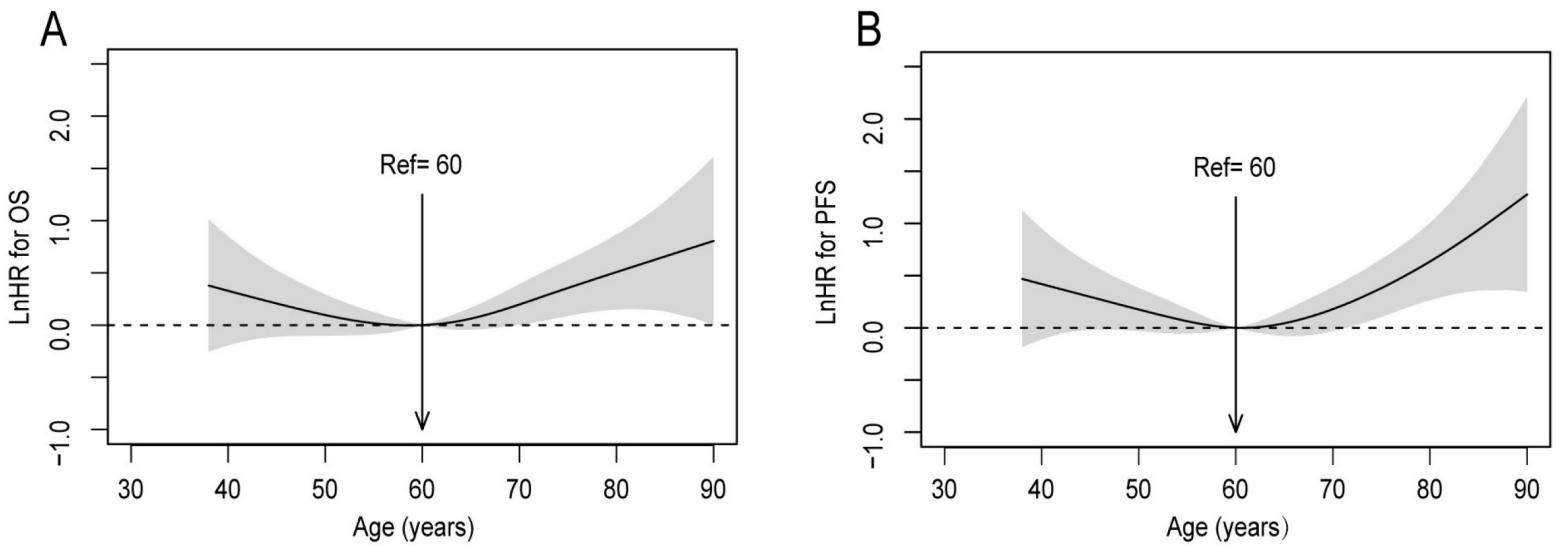
Linear-dependent effect of increasing age on OS and PFS before adjustment. The estimated logarithm HRs (solid line) with 95% CIs (shading) for the association of patients' age with (A) OS, and (B) PFS, in 568 patients with upper ESCC based on the dfmacox in a smoothHR (the optimal extended Cox-type additive hazard regression unadjusted model). The effect of age on the risk of mortality and disease progression was assessed via a penalized spline (P-spline) expansion, with patients' age as a continuous covariate. An age cutoff of 60 years (indicated by the vertical line), was taken as the optimal point for calculating the HR

Investigating the prognostic value of age in patients with upper ESCC, the P-splines model revealed U-shaped survival curves, with 60 years being the optimal cutoff age for differences in OS and PFS (Figure 1A, 1B). The curve of OS tended to be relatively flat, from 50 to 70 years. Consequently, patients were classified into 3 groups based on the smoothHR, as follows: ≤50, 51-69, and ≥70 years (with 81, 408, and 79 subjects, respectively).

**Figure 2:**
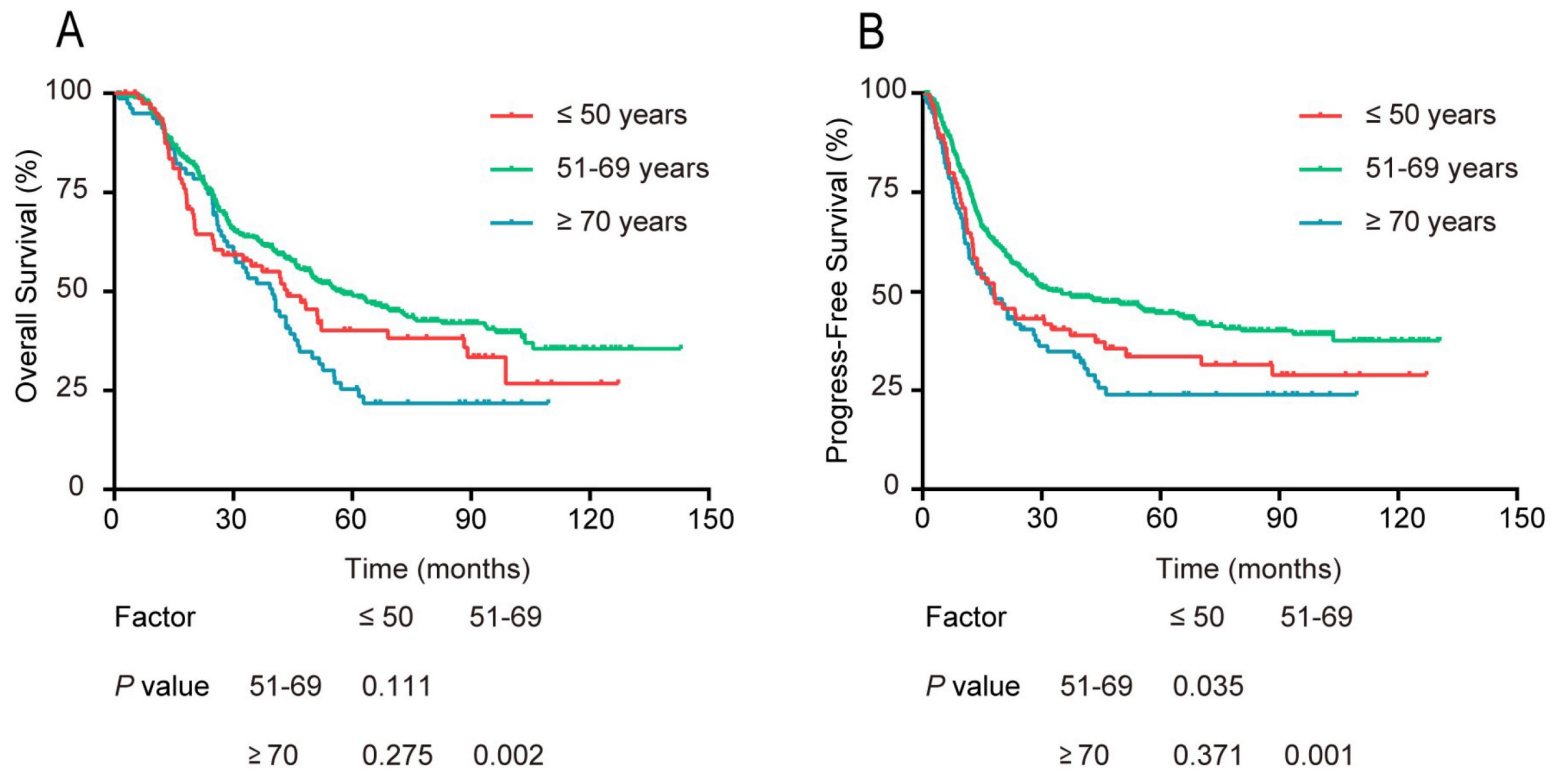
Survival rates of patients stratified into 3 age groups: ≤50, 51-69, and ≥70 years old. (A) OS, and (B) PFS

Comparing the survival differences among the 3 age groups, the univariate survival analysis indicated no significant difference in OS between patients aged 51-69 years and those ≤ 50 years. The PFS curve of the 51-69 year age group was markedly better than that of the ≤ 50 year group (P = 0.035, Figure 2). Concerning OS and PFS, both were significantly better for the 51-69 year group compared with that of the ≥ 70 year group (P = 0.002, P = 0.001). Besides, there was no significant difference in the ≤ 50 year group and ≥ 70 year group in either OS or PFS (P > 0.05).

The multivariate analyses showed no statistically significant differences in either PFS or OS between the groups aged ≤ 50 and 51-69 years. Patients aged ≥ 70 years had a significantly higher risk of mortality and disease progression than did those aged 51-69 years (Table 2).

### Age as an independent prognostic factor for survival after adjustment

**Figure 3:**
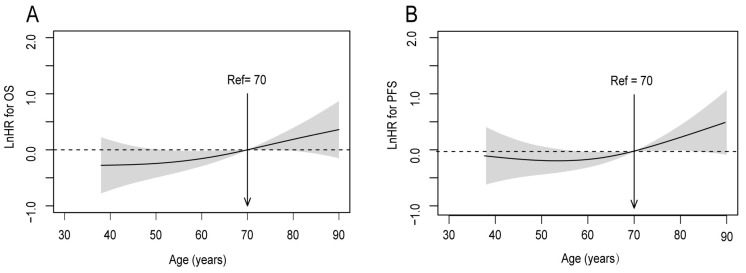
Linear-dependent effect of increasing age on OS and PFS for patients with upper ESCC after adjustment. The model of (A) OS and (B) PFS adjusted for GTVp; gender; lymph node metastasis; clinical T stage; and clinical N stage. An age cutoff of 70 years (indicated by the vertical line) was taken as the optimal point for calculating the HR

To calibrate the potential indication bias, covariates were adjusted by the multivariate Cox proportional hazards regression in smoothHR. The covariates were the following: GTVp; gender; lymph node metastasis; clinical T stage; and clinical N stage. It was observed that the risk (i.e., log hazard ratio, ln[HR]) of mortality and disease progression increased steadily with age, with a particular increase in patients aged ≥ 70 years (Figure 3). Accordingly, in further analyses patients aged ≤ 50 and 51-69 years were combined into one group.

**Figure 4:**
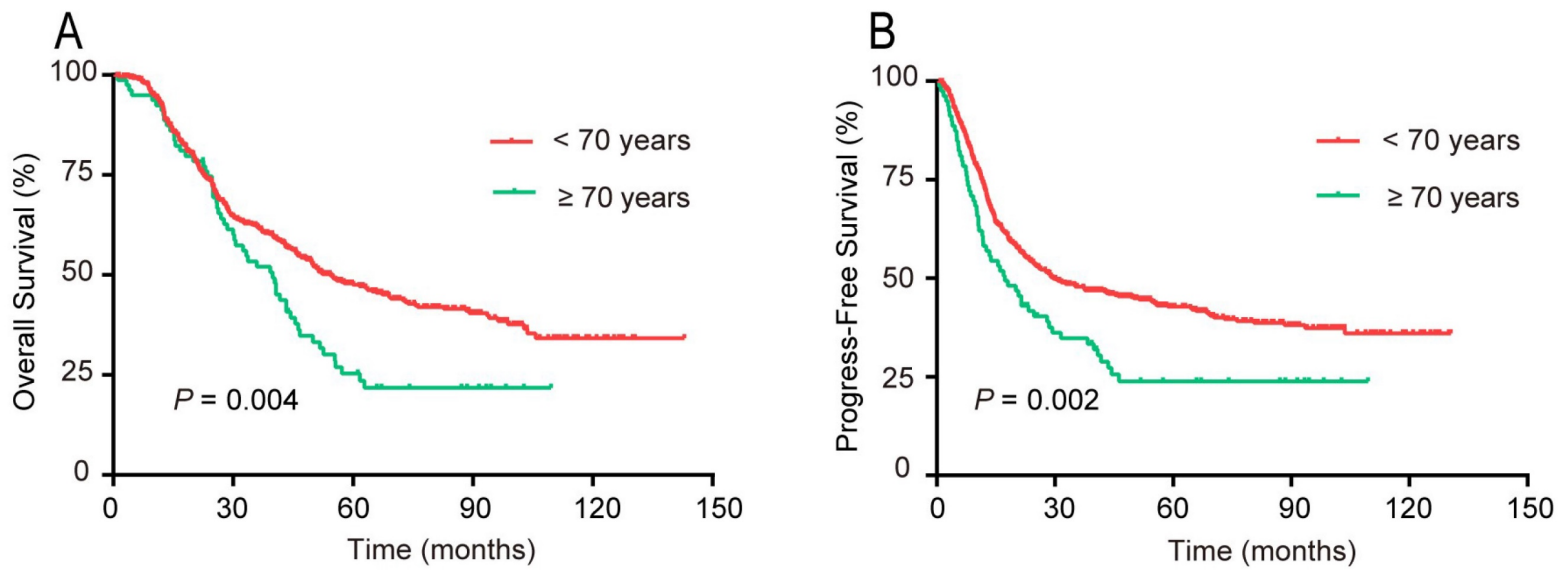
Survival rates of patients with upper ESCC stratified by age into two groups: <70 and ≥70 years. (A) OS; (B) PFS

According to the univariate survival analysis, patients aged ≥70 years had markedly poorer 5-year OS (P = 0.004) and PFS (P = 0.002) rates compared with those aged < 70 years, respectively (Figure 4). The multivariate analysis indicated that age ≥ 70 years was an unfavorable independent prognostic factor (Table 3).

### Effect of age on treatment selection

**Figure 5:**
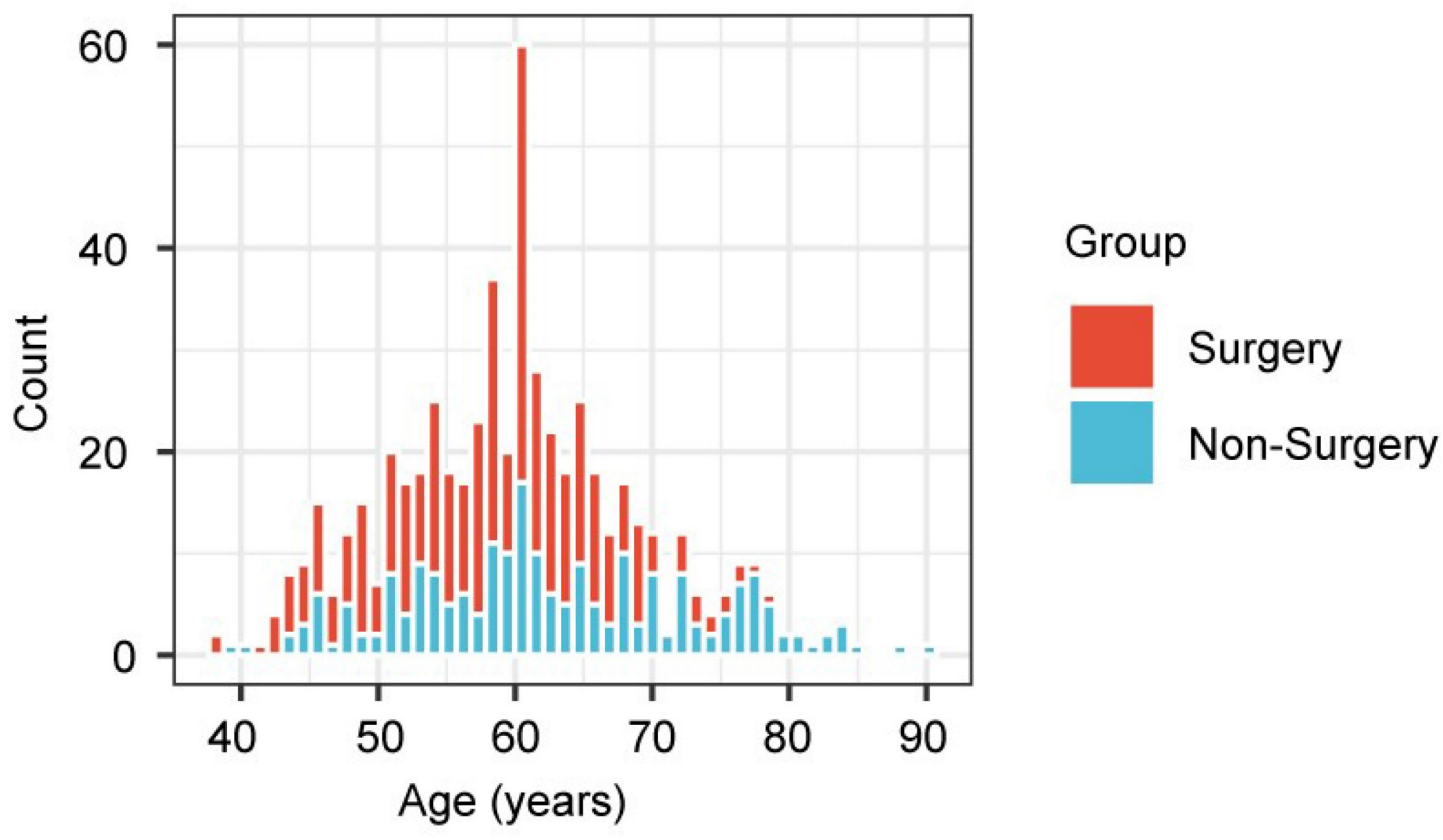
Bar plot representing the proportion of patients, stratified by age, who underwent surgery, compared with non-surgical therapies

This retrospective analysis showed that the treatments of the 2 age groups differed greatly, and therefore there exists the possibility of selection bias (Figure 5). Compared with the younger patients, those aged ≥ 70 years were less likely to undergo surgery, but were treated by non-surgical methods. Therefore, the prognostic effect of treatment was explored for each age group.

**Figure 6:**
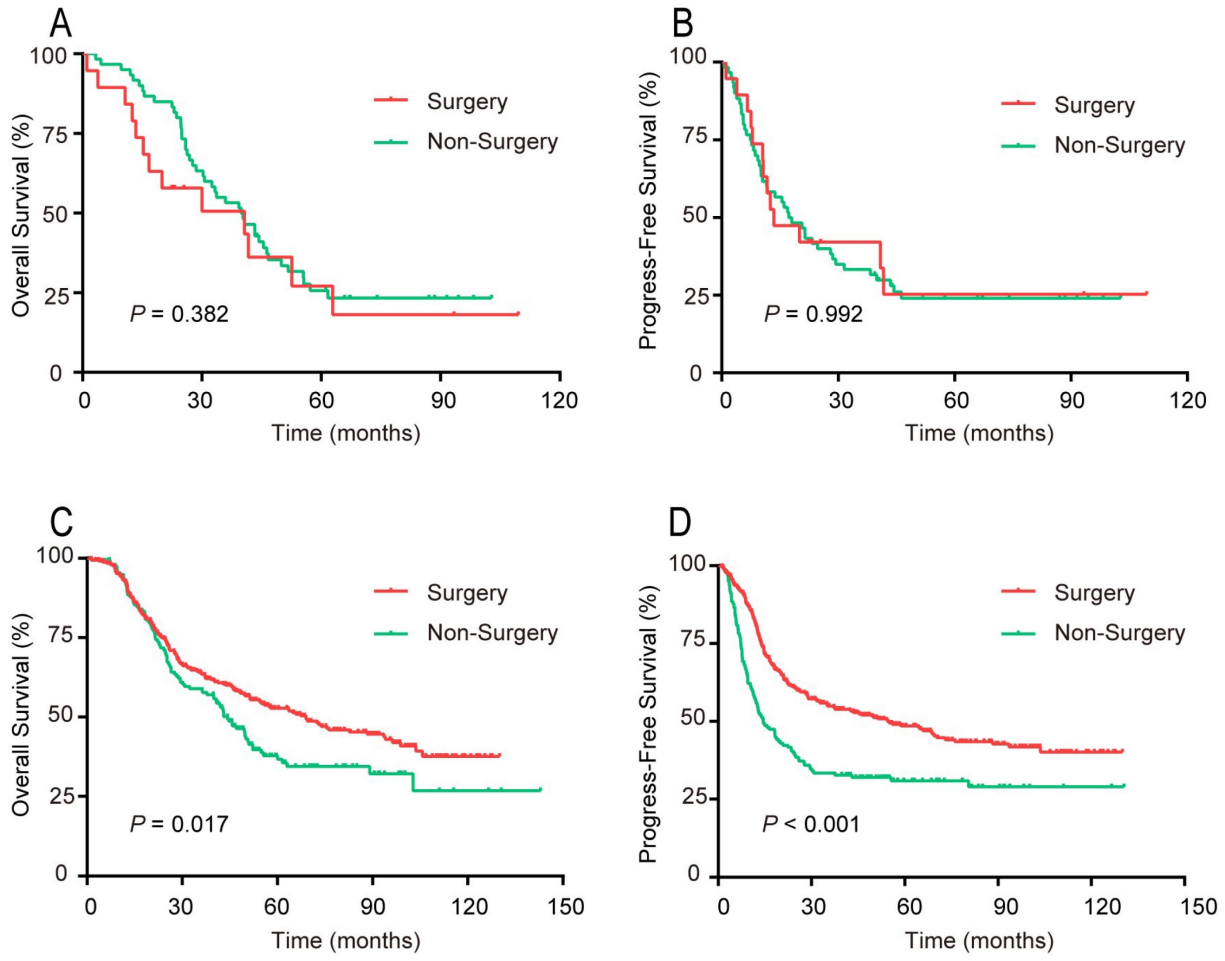
Survival rates of patients in 2 age groups, surgical compared with non-surgical therapy. (A, B) Aged ≥70 years. (A) OS; (B) PFS. (C, D) Aged <70 years. (C) OS; (D) PFS

For patients aged ≥ 70 years, the OS and PFS curves, respectively, did not differ significantly between the surgery and non-surgery groups (Figure 6). For patients younger than 70 years, both the OS and PFS curves of the surgery group were noticeably preferable to that of the non-surgery group.

## Discussion

To our best knowledge, this study has the largest population with upper ESCC to explore the importance of age to prognosis and indication of treatment. The initial 3 age groups considered (≤ 50, 51-69, and ≥ 70 years) was based on a cutoff of 60 years for distinguishing survival differences before adjustment. However, the multivariate Cox regression showed that the OS and PFS of patients aged ≤ 50 years did not differ significantly from that of the 51-69 year group. After adjustment for covariates, P-spline regression confirmed an age-dependent effect, with 70 years as an optimal cutoff age for survival differences. It was further verified that age ≥ 70 years was an adverse independent factor for predicting OS and PFS, and that within this age range, surgery provided no survival benefit relative to non-surgical methods. However, for patients younger than 70 years, the OS and PFS of those who received surgery was significantly longer than that of patients given only non-surgical options. These results indicate that age is a predictive factor for prognosis of patients with upper ESCC, and can aid treatment strategies.

Before adjustment, the P-spline regression analysis suggested that an optimal cutoff age for survival was 60 years. The univariate survival analysis showed that the PFS curve of patients aged 51 to 69 years was slightly better than that of patients aged up to 50 years. However, according to the succeeding multivariate survival analyses, there was no statistically significant difference in either PFS or OS between the ≤ 50 and 51-69 year age groups. In this study, age was treated as a continuous variable when analyzed by Cox proportional hazards regression, via P-splines in smoothHR. This should be a more accurate reflection of the effect of age on patients' outcome, compared with analyzing age in ranges. As in previous studies 23,24, the log-rank test was used to determine the prognostic value of age with better accuracy. Furthermore, multivariate Cox regression analysis was performed to eliminate non-significant explanatory variables 6,16,25 and validate the independent prognostic value of age. Finally, the present study found that patients aged ≥ 70 years had a worse prognosis than did those aged 51 to 69 years. This is consistent with another study 26, and suggests that advanced age at the time of EC diagnosis is associated with a worse prognosis, compared with younger patients. Taken together, P-spline regression analysis, plus univariate and then multivariate survival analyses, seems to be a perfect procedure to initially evaluate the prognostic significance of age as a continuous variable.

After adjusting for other clinical covariates, P-spline regression analysis confirmed an age-dependent effect in upper ESCC, and the risk of mortality and disease progression increased with age, especially for patients aged ≥ 70 years. By further univariate and multivariate analyses, ≥ 70 years was identified as a negative factor for independently predicting OS and PFS. Hence, the present study is the first to investigate the influence of age on prognosis using multivariate P-spline regression to calibrate the potential indication bias in patients with upper ESCC. Recently, several studies have focused on associations between patient age and survival prognosis in EC 27-29. However, there is still no definitive conclusion regarding an optimal cutoff age to differentiate survival rates. Nakashima et al. 29 determined that patients with EC who were 65 years and older experienced significantly worse surgical outcomes. Lagergren et al. 1 reported that age ≥ 75 years at the time of esophagectomy for EC was an independent risk factor for higher short- and longer-term mortality. Bakhos et al. 30 even noted that age ≥80 years was an independent indicator predicting 30-day mortality after esophagectomy. The above postulated cutoff ages mainly relied on clinical experience. In the current study using P-splines in smoothHR, 70 years was the determined optimal cutoff age. This age appeared more reasonable, as it was based on an association between age as a continuous variable and survival rates.

It is a common knowledge that older patients with EC generally receive less aggressive treatment when compared with their younger counterparts. In the present study, the ages of the surgery and non-surgery groups were markedly different. To explore further the prognostic effect, the 2 treatment groups were stratified by age. For patients aged ≥ 70 years, the PFS and OS rates did not differ significantly between the 2 treatment groups. This may be because patients at these ages often have a poorer prognosis due to comorbidities and higher rates of complications, regardless of treatment. In a retrospective study, Faiz et al. 31 reported that for patients with ESCC with ≥ 2 comorbidities, or age ≥ 75 years, definitive chemoradiotherapy may be the preferred treatment modality. In another study, radiotherapy was found crucial for the curative management of inoperable elder patients with ESCC, significantly improving locoregional control and prolonging survival 32. Therefore, caution is warranted when administering aggressive treatment to elderly patients, due to their poor physical condition.

For patients aged younger than 70 years, however, the surgery group had significantly better 5-year OS and PFS rates than did the non-surgery group. As patients in this age range can generally tolerate surgery, it considered the first option; exceptions are patients with non-metastatic upper ESCC staged as T4b 33, or those who are inoperable because of large local invasion of adjacent anatomical structures. For operable patients with EC, surgery has a better therapeutic effect than non-surgery 34,35. Neoadjuvant chemoradiotherapy is recommended by the 2019 NCCN guidelines 14 as the first treatment choice for operable patients with ESCC with locally advanced disease, based on 2 large, prospective, randomized CROSS clinical trials 36,37. Hence, it is not difficult to understand that patients younger than 70 years treated with surgery have a better prognosis compared with those given non-surgical treatment only. For nonresectable patients with upper ESCC at first diagnosis, more aggressive strategies prior to surgery must be explored.

Despite the large upper ESCC cohort of the present study, its retrospective nature makes patient selection and confounding bias inevitable. A large, prospective, and multicentral study is warranted to validate that age as a continuous variable has prognostic value in upper ESCC. This study is also limited in that geriatric assessment data were unavailable, and as such interactions of age-associated comorbidities and treatments could not be discussed well. Nevertheless, comorbidities are acknowledged risk factors of prognosis for elderly patients with ESCC 34,38,39. Lastly, this analysis did not incorporate other independent prognostic factors, such GTVp, gender, clinical T stage, and clinical N stage, which may contribute greatly to improving prognostic accuracy and treatment options. A nomogram model is greatly needed for the best prediction of survival of patients with upper ESCC.

## Conclusion

Taken together, this study determined that age was an independent factor for predicting OS and PFS in a cohort of patients with upper ESCC. For patients aged 70 years and older, there was no significant survival benefit associated with surgery compared with non-surgical therapies. For patients younger than 70 years, surgery was the more effective treatment. These findings may help clinicians strategize individualized treatment for patients with upper ESCC.

## Figures and Tables

**Table 1:** Patient characteristics by age group

Characteristics	All patients	Age groups (years)			*P* value
		Age ≤50	Age 51-69	Age ≥70	
	No. (%)	No. (%)	No. (%)	No. (%)	
Total	568(100)	81(14.3)	408(71.8)	79(13.9)	
**Gender**					0.001
Male	391(68.8)	65(80.2)	284(69.6)	42(53.2)	
Female	177(31.2)	16(19.8)	124(30.4)	37(46.8)	
**GTV-p**					0.012
≤30 cm^3^	396(69.7)	50(61.7)	299(73.3)	47(59.5)	
>30 cm^3^	172(30.3)	31(38.3)	109(26.7)	32(40.5)	
**LNM**					0.097
No	252(44.4)	27(33.3)	188(46.1)	37(46.8)	
Yes	316(55.6)	54(66.7)	220(53.9)	42(53.2)	
**cT stage**					0.447
T1	15(2.6)	2(2.5)	11(2.7)	2(2.5)	
T2	112(19.7)	16(19.8)	87(21.3)	9(11.4)	
T3	247(43.5)	33(40.7)	179(43.9)	35(44.3)	
T4	194(34.2)	30(37.0)	131(32.1)	33(41.8)	
**cN stage**					0.003
N0	285(50.2)	36(44.4)	213(52.2)	36(45.6)	
N1	185(32.6)	27(33.3)	140(34.3)	18(22.8)	
N2	88(15.5)	16(19.8)	48(11.8)	24(30.4)	
N3	10(1.8)	2(2.5)	7(1.7)	1(1.3)	
**cTNM stage**					0.330
I-II	261(46.0)	33(40.7)	198(48.5)	30(38.0)	
III	117(20.6)	17(21.0)	83(20.3)	17(21.5)	
IV	190(33.5)	31(38.3)	127(31.1)	32(40.5)	
**Tumor length**					0.787
≤5 cm	363(63.9)	49(60.5)	263(64.5)	51(64.6)	
>5 cm	205(36.1)	32(39.5)	145(35.5)	28(35.4)	
**Treatment**					< 0.001
Surgery	352(62.0)	58(71.6)	275(67.4)	19(24.1)	
Non-Surgery	216(38.0)	23(28.4)	133(32.6)	60(75.9)	

GTVp, primary gross tumor volume; LNM, lymph node metastasis.

**Table 2:** Univariate and multivariate analyses of risk factors influencing OS and PFS for patients with upper ESCC

Characteristics	OS		PFS		OS		PFS	
	95% CI	*P* value	95% CI	*P* value	HR 95% CI	*P* value	HR 95% CI	*P* value
Age (years)								
≤50 vs 51-69	0.947~1.771	0.106	1.021~1.857	0.036	1.072 0.780~1.472	0.668	1.153 0.852~1.560	0.355
≥70 vs 51-69	1.190~2.138	0.002	1.223~2.178	0.001	1.605 1.170~2.203	0.003	1.479 1.098~1.991	0.010
Gender	0.494~0.820	< 0.001	0.543~0.883	0.003	0.677 0.522~0.878	0.003	0.744 0.579~0.956	0.021
GTV-p	1.625~2.550	< 0.001	1.738~2.693	< 0.001	1.703 1.326~2.186	< 0.001	1.637 1.291~2.074	< 0.001
LNM	1.615~2.581	< 0.001	1.671~2.623	< 0.001	1.799 1.414~2.290	<0.001	1.602 1.242~2.068	< 0.001
cT stage	1.255~1.685	< 0.001	1.285~1.710	< 0.001	1.249 1.062~1.469	0.007	1.180 1.014~1.374	0.032
cN stage	1.307~1.691	< 0.001	1.439~1.860	< 0.001	-	-	1.243 1.067~1.448	0.005
Treatment	0.582~0.908	0.005	0.461~0.707	< 0.001	-	-	-	-

CI, confidence interval; HR, hazard ratio.

**Table 3:** Survival analyses of risk factors influencing OS and PFS for patients with upper ESCC

Characteristics	OS		PFS		OS		PFS	
	95% CI	*P* value	95% CI	*P* value	HR 95% CI	*P* value	HR 95% CI	*P* value
Age (years) (<70 vs ≥70)	1.146~2.037	0.004	1.166~2.053	0.003	1.472 1.095~1.979	0.010	1.443 1.077~1.934	0.014
Gender	0.494~0.820	< 0.001	0.543~0.883	0.003	0.666 0.514~0.863	0.002	0.738 0.575~0.947	0.017
GTV-p	1.625~2.550	< 0.001	1.738~2.693	< 0.001	1.608 1.269~2.038	< 0.001	1.641 1.295~2.078	< 0.001
LNM	1.615~2.581	< 0.001	1.671~2.623	< 0.001	1.777 1.399~2.257	< 0.001	1.619 1.256~2.086	< 0.001
cT stage	1.255~1.685	< 0.001	1.285~1.710	< 0.001	1.212 1.038~1.416	0.015	1.181 1.015~1.375	0.032
cN stage	1.307~1.691	< 0.001	1.439~1.860	< 0.001	-	-	1.244 1.068~1.449	0.005
Treatment	0.582~0.908	0.005	0.461~0.707	< 0.001	-	-	-	-

## Abbreviations

ESCC: esophageal squamous cell carcinoma
P-spline: penalized spline
OS: overall survival
PFS: progression-free survival
EC: esophageal cancer
NCCN: National Comprehensive Cancer Network
GTVp: primary gross tumor volume
ECOG/PS: Eastern Cooperative Oncology Group performance status
UICC/AJCC: Union for International Cancer Control/American Joint Committee on Cancer
CT: computed tomography
PET: positron emission tomography
HR: hazard ratio
